# Crystal structure of an unknown tetra­hydro­furan solvate of tetra­kis­(*μ*_3_-cyanato-κ^3^ *N*:*N*:*N*)tetra­kis­[(triphenyl­phosphane-κ*P*)­silver(I)]

**DOI:** 10.1107/S2056989015017636

**Published:** 2015-09-30

**Authors:** Peter Frenzel, Dieter Schaarschmidt, Alexander Jakob, Heinrich Lang

**Affiliations:** a Technische Universität Chemnitz, Faculty of Natural Sciences, Institute of Chemistry, Inorganic Chemistry, 09107 Chemnitz, Germany; b University of Regensburg, Institute of Organic Chemistry, Universitätsstrasse 31, 93040 Regensburg, Germany

**Keywords:** crystal structure, silver(I), cyanate ligand, phosphine ligand, Ag_4_N_4_ heterocubane, SQUEEZE procedure

## Abstract

In the title compound, [{[(C_6_H_5_)_3_P]Ag}_4_{NCO}_4_], a distorted Ag_4_N_4_-heterocubane core is set up by four Ag^I^ ions being coordinated by the N atoms of the cyanato anions in a *μ*_3_-bridging mode. In addition, a tri­phenyl­phosphine ligand is datively bonded to each of the Ag^I^ ions. Intra­molecular Ag⋯Ag distances as short as 3.133 (9) Å suggest the presence of argentophilic (*d* ^10^⋯*d* ^10^) inter­actions. Five moderate-to-weak C—H⋯O hydrogen-bonding inter­actions are observed in the crystal structure, spanning a three-dimensional network. A region of electron density was treated with the SQUEEZE procedure in *PLATON* [Spek (2015). *Acta Cryst.* C**71**, 9–18] following unsuccessful attempts to model it as being part of disordered tetra­hydro­furan solvent mol­ecules. The given chemical formula and other crystal data do not take into account these solvent mol­ecules.

## Abstract 2

In the title compound a distorted Ag_4_N_4_-heterocubane core is set up by Ag^I^ cations and N atoms of cyanate anions. The core is decorated by four tri­phenyl­phosphine ligands bonded to the Ag^I^ cations. Ag⋯Ag distances as short as 3.133 (9) Å suggest the presence of argentophilic (*d* ^10^⋯*d* ^10^) inter­actions.

## Chemical context   

A large number of studies about silver precursors, for instance silver(I) carboxyl­ates, silver(I) di­thio­carbamates or silver(I) β-diketonates have been reported, due to their suitability in manifold application methods such as CCVD (combustion chemical vapour deposition) or CVD (chemical vapour deposition) processes (Struppert *et al.*, 2010 ▸; Schmidt *et al.*, 2005 ▸; Haase *et al.*, 2005*a* ▸,*b* ▸; Lang & Buschbeck, 2009 ▸; Lang, 2011 ▸; Lang & Dietrich, 2013 ▸; Jakob *et al.*, 2006 ▸; Chi *et al.*, 1996 ▸; Chi & Lu, 2001 ▸), inkjet printing (Jahn *et al.*, 2010 ▸), joining processes (Oestreicher *et al.*, 2013 ▸), their use as single-source precursors for Ag_2_S formation (Mothes *et al.*, 2015*a* ▸,*b* ▸), catalysis (Steffan *et al.*, 2009 ▸) and self-assembly of silver nanoparticles (Bokhonov *et al.*, 2014 ▸).
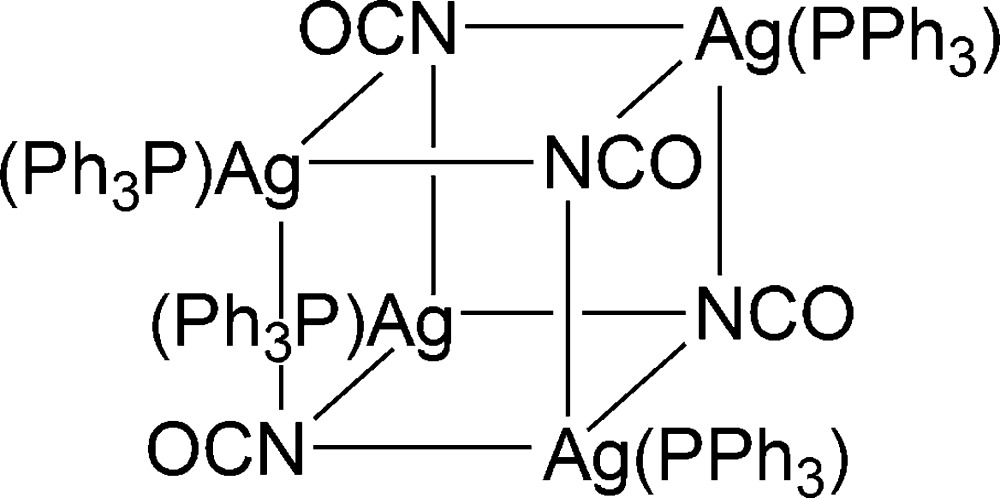


In contrast, hardly any research has been done on compounds such as metal alkyl allophanates. Despite the inter­esting features of this type of compounds, only few research groups have so far been involved in the synthesis (Clusius & Endtinger, 1960 ▸; Becker & Eisenschmidt, 1973 ▸; Dains & Wertheim, 1920 ▸) and further modification of this family of compounds (Kawakubo *et al.*, 2015 ▸; Potts *et al.*, 1990 ▸; Bachmann & Maxwell, 1950 ▸; Murray & Dains, 1934 ▸; Biltz & Jeltsch, 1923 ▸). To the best of our knowledge, two synthetic approaches for the preparation of potassium and silver salts of ethyl allophanate have been described in the literature (Blair, 1926 ▸; Dains *et al.*, 1919 ▸). The identity of metal allophanates has been confirmed by elemental analysis. For the application of these precursors, full characterization and the investigation of their thermal behaviour is required. In the context of precursor design for MOD (metal organic deposition) inks, we are inter­ested in the synthesis, characterization and application of such complexes for inkjet printing.

To get access to a large range of metal allophanates (*e.g.* Cu, Ni or Zn), a modified synthetic procedure with respect to the method reported by Dains *et al.* (1919 ▸) was applied for the synthesis of silver allophanates among others. The initial step involved conversion of ethyl allophanate with sodium ethano­late for use of the resulting solid in a further reaction to form the respective silver complex. To analyse the sparingly soluble compound, IR spectroscopy has been applied. A comparison of the measured spectrum with that of ethyl allophanate showed the absence of the carbonyl band at 1701 cm^−1^ and the appearance of a new band at 2170 cm^−1^ of high intensity, indicating the formation of silver iso­cyanate (Ellestad *et al.*, 1972 ▸). To confirm the assumption of the formation of silver iso­cyanate, the respective solid was treated with tri­phenyl­phosphine (PPh_3_) in tetra­hydro­furan (THF) and subsequently crystallized. The characterization of the crystals obtained by X-ray diffraction, NMR and IR spectroscopy is in accordance with the formation of the title compound, [{((C_6_H_5_)_3_P)Ag}_4_{NCO}_4_], (I).

## Structural commentary   

**Figure 1:**
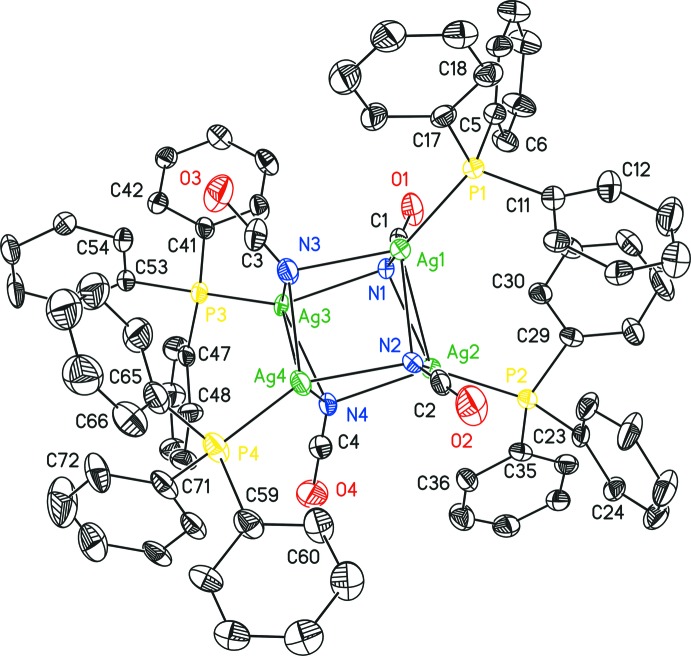
The mol­ecular structure of (I) with displacement ellipsoids drawn at the 30% probability level. Hydrogen atoms and the minor parts of the disordered atoms are omitted for clarity.

The title compound consists of a Ag_4_N_4_-heterocubane core formed by κ*N*-coordination of four cyanate anions towards four Ag^I^ cations in a *μ*_3_-bridging mode (Fig. 1 ▸). Each Ag^I^ cation is additionally coordinated by a PPh_3_ ligand. Disorder is observed in the crystal structure of (I) affecting the Ag3 and Ag4 sites, together with their bonded PPh_3_ moieties. However, the respective components of both disordered Ag(PPh_3_) units share one phenyl ring (C41–C46 and C59–C64). The Ag_4_N_4_-heterocubane is distorted which is reflected by the variation of the Ag—N distances in the range 2.273 (13)–2.605 (12) Å. Likewise, the Ag—N—Ag [78.7 (3) – 98.5 (3)°] and N—Ag—N [80.9 (3) – 98.5 (3)°] angles significantly deviate from 90°. The Ag_2_N_2_-faces of the Ag_4_N_4_-core are not planar [r.m.s. deviations in the range 0.0293 (Ag1, Ag4, N2, N3) to 0.1947 Å (Ag3, Ag4′, N3, N4)], however, the opposing least-squares planes are almost parallel [angles between planes: 0.40 (3) and 3.2 (3)°]. Opposing planes are twisted by some degrees relative to each other, which is reflected by the Ag—N—Ag—N and N—Ag—N—Ag torsion angles ranging from 2.8 (3)–19.4 (3)°. As a result of the distortion of the Ag_4_N_4_-core, the Ag⋯Ag and N⋯N separations differ significantly. The shortest distances are observed between Ag1 and Ag2 as well as Ag3/Ag3′ and Ag4/Ag4′ (Table 1 ▸). Considering the contact radius of silver (1.72 Å; Bondi, 1964 ▸) a weak argentophilic inter­action between these pairs of atoms is most likely (Schmidbaur & Schier, 2015 ▸). The Ag—P separations [2.336 (15)–2.39 (2) Å] are characteristic for an Ag^I^(PPh_3_) fragment. The scattering contributions of two severely disordered THF solvent mol­ecules were treated with the SQUEEZE procedure in *PLATON* (Spek, 2015 ▸). The calculated electron count of 350 electrons per unit cell is in good agreement with the composition of (I)·2THF. In contrast, NMR analysis of the crystals after deca­ntation of the supernatant solvent and drying *in vacuo* reveals a composition of (I)·0.25THF. This discrepancy may be due to a facile evaporation of the co-crystallized solvent under reduced pressure.

## Supra­molecular features   

Five moderate-to-weak C—H⋯O hydrogen bonds (Steiner, 2002 ▸) are observed in the crystal structure of (I) (Table 2 ▸). Four of those participate in the formation of a three-dimensional network. No obvious π–π-stacking inter­actions between the phenyl rings are present.

## Database survey   

There are 75 structures of Ag_4_ *E*_4_-heterocubanes (*E* = N, O, Cl, Br, I) in the CSD database (Groom & Allen, 2014 ▸; CSD Version 5.36); in 35 of these complexes, silver is coordinated by phospho­rus. Ag_4_N_4_-heterocubanes are relatively rare as there are only three examples known so far (Bowmaker *et al.*, 1998 ▸; Partyka & Deligonul, 2009 ▸). These include the tri­cyclo­hexyl­arsine analogue of (I) as well as its pyridine solvate (Bowmaker *et al.*, 1998 ▸). All reported Ag_4_N_4_-heterocubanes are less distorted than (I), which is reflected in a much less pronounced deviation of the Ag⋯Ag distances in the heterocubane. A *μ*_3_-κ*N* coordination of the cyanate anions towards Ag^I^ has been described for five compounds only (Bowmaker *et al.*, 1998 ▸; Di Nicola *et al.*, 2005 ▸, 2006 ▸). The average Ag—N distance in these compounds (2.433 Å) is in good agreement with the corresponding value of 2.408 Å in (I).

## Synthesis and crystallization   

To a solution of sodium ethano­late in ethanol, generated *in situ* by dissolving sodium (349 mg, 15.2 mmol) in anhydrous ethanol (40 ml), was added at 273 K ethyl allophanate (1.92 g, 14.5 mmol). The reaction was heated to reflux overnight. The colourless precipitate formed was filtered off, washed thrice with ethanol (each 20 ml) and dried under vacuum (yield: 850 mg). The resulting solid material (407 mg) was dissolved in water (20 ml) and was added dropwise to a solution of silver nitrate (449 mg, 2.64 mmol) in water (15 ml). The suspension obtained was stirred at ambient temperature overnight. Filtration afforded a colourless solid, which was washed with cold water (20 ml) and dried under vacuum (yield: 250 mg). A suspension of this solid (120 mg) in anhydrous THF (20 ml) was treated with PPh_3_ (265 mg, 1.01 mmol) at 273 K. After stirring overnight at this temperature, the reaction mixture was filtered through a pad of celite. Removal of all volatiles under reduced pressure afforded a pale purple solid (yield: 313 mg, 0.189 mmol, 95% based on [AgNCO]). Colourless crystals of (I) were obtained by slow diffusion of diethyl ether into a THF solution of (I) at ambient temperature.

M.p. 458 K (decomp.). ^1^H NMR (500 MHz, CDCl_3_, 298 K, ppm): *δ* = 7.40–7.28 (*m*, 60H, C_6_H_5_). ^13^C{^1^H} NMR (126 MHz, CDCl_3_, 298 K, p.p.m.): *δ* = 134.0 (*d*, 2C, ^2^ *J*_PC_ = 16.5 Hz, C_6_H_5_), 132.1 (*d*, 1C, ^1^ *J*_PC_ = 27.3 Hz, C_6_H_5_), 130.4 (*d*, 1C, ^4^ *J*_PC_ = 1.5 Hz, C_6_H_5_), 129.1 (*d*, 2C, ^3^ *J*_PC_ = 9.8 Hz, C_6_H_5_). The resonance signal of the cyanate carbon atom is not observed under the measurement conditions applied. ^31^P{^1^H} NMR (203 MHz, CDCl_3_, 298 K, p.p.m.): *δ* = 9.0 (*s*). IR (KBr, cm^−1^): *ν* = 3449 (*w*), 3356 (*w*), 2170 (*vs*, N=C=O), 1603 (*w*), 1429 (*w*), 1388 (*w*), 1300 (*m*), 1206 (*m*), 638 (*m*).

## Refinement details   

**Figure 2:**
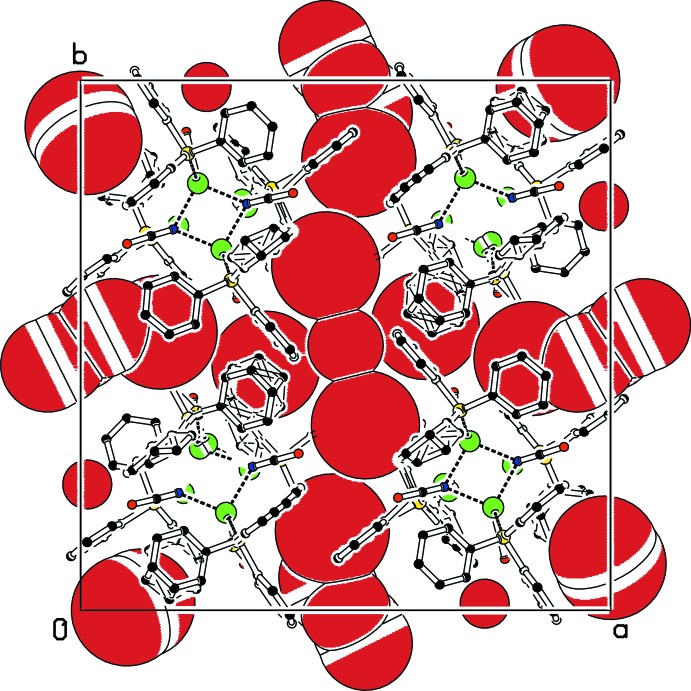
Packing diagram of (I) viewed along [001]. Voids in the structure are represented by red spheres (drawn using the CAVITYPLOT routine in *PLATON*; Spek, 2009 ▸). Hydrogen atoms were omitted for clarity. Dashed lines represent coordinative bonds. Colour code: black (C), red (O), yellow (P), green (Ag).

Crystal data, data collection and structure refinement details are summarized in Table 3 ▸. In the final refinement of (I) thirteen reflections, *viz.* (240), (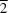
60), (040), (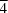
42), (032), (302), (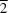
40), (222), (250), (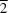
22), (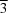
11), (340), and (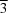
21), were omitted owing to poor agreements between observed and calculated intensities. C-bonded H atoms were placed in calculated positions and constrained to ride on their parent atoms, with *U*_iso_(H) = 1.2*U*_eq_(C) and a C—H distance of 0.93 Å. Atoms Ag3 and Ag4 and two of the four P atoms of the PPh_3_ moieties with attached phenyl rings are disordered over two sets of sites, with occupancy ratios of 0.54 (4):0.46 (4) and 0.55 (2):0.45 (2), respectively. A phenyl ring of another PPh_3_ moiety is likewise disordered over two sets of sites in a 0.67 (5):0.33 (5) ratio. The disordered phenyl rings were treated by rigid-group refinements. If necessary, the respective C—P distances were restrained to 1.85 (2) Å. Anisotropic displacement parameters of all atoms were restrained using enhanced rigid-bond restraints (RIGU command, esds 0.004 Å^2^; Thorn *et al.*, 2012 ▸). Solvent contributions to the scattering have been removed using the SQUEEZE procedure (Spek, 2015 ▸) in *PLATON* (Spek, 2009 ▸). SQUEEZE calculated a void volume of approximately 2494 Å^3^ occupied by 350 electrons per unit cell which points to the presence of two THF mol­ecules per formula unit. Fig. 2 ▸ shows the positions of the voids within the unit cell.

## Supplementary Material

Crystal structure: contains datablock(s) I, New_Global_Publ_Block. DOI: 10.1107/S2056989015017636/wm5201sup1.cif

Structure factors: contains datablock(s) I. DOI: 10.1107/S2056989015017636/wm5201Isup2.hkl

CCDC reference: 1426238

Additional supporting information:  crystallographic information; 3D view; checkCIF report

## Figures and Tables

**Table 1:** AgAg and NN separations ()

Ag3Ag4	3.133(9)	Ag1Ag3	3.605(8)
Ag3Ag4	3.156(8)	Ag2Ag4	3.615(8)
Ag1Ag2	3.1906(10)	Ag1Ag4	3.616(9)
Ag3Ag4	3.215(8)	N1N3	3.210(10)
Ag3Ag4	3.250(9)	N2N3	3.213(9)
Ag2Ag4	3.428(10)	N1N4	3.220(9)
Ag1Ag4	3.461(8)	N2N4	3.247(10)
Ag1Ag3	3.494(8)	N1N2	3.572(11)
Ag2Ag3	3.523(6)	N3N4	3.599(14)
Ag2Ag3	3.545(5)		

**Table 2:** Hydrogen-bond geometry (, )

*D*H*A*	*D*H	H*A*	*D* *A*	*D*H*A*
C9H9O2^i^	0.93	2.37	3.177(12)	145
C16H16O2	0.93	2.59	3.324(17)	136
C25H25O1^ii^	0.93	2.48	3.358(12)	157
C51H51O4^iii^	0.93	2.22	3.07(2)	151
C67H67O3^iv^	0.93	2.19	3.01(2)	147

Symmetry codes: (i) 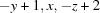; (ii) 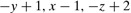; (iii) 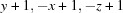; (iv) 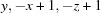.

**Table 3:** Experimental details

Crystal data	
Chemical formula	[Ag_4_(CNO)_4_(C_18_H_15_P)_4_]
*M*_r_	1648.64
Crystal system, space group	Tetragonal, *P* 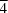
Temperature (K)	110
*a*, *c* ()	24.0846(3), 15.2037(3)
*V* (^3^)	8819.2(3)
*Z*	4
Radiation type	Mo *K*
(mm^1^)	0.99
Crystal size (mm)	0.35 0.30 0.20

Data collection	
Diffractometer	Oxford Gemini S diffractometer
Absorption correction	Multi-scan (*CrysAlis RED*; Oxford Diffraction, 2006 ▸)
*T*_min_, *T*_max_	0.912, 1.000
No. of measured, independent and observed [*I* > 2(*I*)] reflections	105239, 20082, 12667
*R*_int_	0.048
(sin /)_max_ (^1^)	0.674

Refinement	
*R*[*F* ^2^ > 2(*F* ^2^)], *wR*(*F* ^2^), *S*	0.045, 0.131, 1.01
No. of reflections	20082
No. of parameters	1018
No. of restraints	1206
H-atom treatment	H-atom parameters constrained
_max_, _min_ (e ^3^)	0.68, 1.88
Absolute structure	Flack *x* determined using 5170 quotients [(*I* ^+^)(*I* )]/[(*I* ^+^)+(*I* )] (Parsons Flack, 2004 ▸)
Absolute structure parameter	0.023(9)

Computer programs: *CrysAlis CCD* and *CrysAlis RED* (Oxford Diffraction, 2006 ▸), *SHELXS2013* and *SHELXTL* (Sheldrick, 2008 ▸), *SHELXL2013* (Sheldrick, 2015 ▸), *ORTEP-3 for Windows* and *WinGX* (Farrugia, 2012 ▸), *PLATON* (Spek, 2009 ▸), SQUEEZE (Spek, 2015 ▸) and *publCIF* (Westrip, 2010 ▸).

## supplementary crystallographic information

### Crystal data

**Table:**

[Ag_4_(CNO)_4_(C_18_H_15_P)_4_]	*D*_x_ = 1.242 Mg m^−^^3^
*M**_r_* = 1648.64	Mo *K*α radiation, λ = 0.71073 Å
Tetragonal, *P*4	Cell parameters from 20599 reflections
*a* = 24.0846 (3) Å	θ = 3.3–28.0°
*c* = 15.2037 (3) Å	µ = 0.99 mm^−^^1^
*V* = 8819.2 (3) Å^3^	*T* = 110 K
*Z* = 4	Block, colourless
*F*(000) = 3296	0.35 × 0.30 × 0.20 mm

### Data collection

**Table:**

Oxford Gemini S diffractometer	*R*_int_ = 0.048
ω scans	θ_max_ = 28.6°, θ_min_ = 2.9°
Absorption correction: multi-scan (*CrysAlis RED*; Oxford Diffraction, 2006)	*h* = −30→31
*T*_min_ = 0.912, *T*_max_ = 1.000	*k* = −30→32
105239 measured reflections	*l* = −19→18
20082 independent reflections	50 standard reflections every 10 reflections
12667 reflections with *I* > 2σ(*I*)	intensity decay: none

### Refinement

**Table:**

Refinement on *F*^2^	Hydrogen site location: inferred from neighbouring sites
Least-squares matrix: full	H-atom parameters constrained
*R*[*F*^2^ > 2σ(*F*^2^)] = 0.045	*w* = 1/[σ^2^(*F*_o_^2^) + (0.0659*P*)^2^ + 1.5343*P*] where *P* = (*F*_o_^2^ + 2*F*_c_^2^)/3
*wR*(*F*^2^) = 0.131	(Δ/σ)_max_ = 0.001
*S* = 1.01	Δρ_max_ = 0.68 e Å^−^^3^
20082 reflections	Δρ_min_ = −1.88 e Å^−^^3^
1018 parameters	Absolute structure: Flack *x* determined using 5170 quotients [(*I*^+^)–(*I*^–^)]/[(*I*^+^)+(*I*^–^)] (Parsons & Flack, 2004)
1206 restraints	Absolute structure parameter: −0.023 (9)

### Special details

**Table:**

Geometry. All e.s.d.'s (except the e.s.d. in the dihedral angle between two l.s. planes) are estimated using the full covariance matrix. The cell e.s.d.'s are taken into account individually in the estimation of e.s.d.'s in distances, angles and torsion angles; correlations between e.s.d.'s in cell parameters are only used when they are defined by crystal symmetry. An approximate (isotropic) treatment of cell e.s.d.'s is used for estimating e.s.d.'s involving l.s. planes.

### Fractional atomic coordinates and isotropic or equivalent isotropic displacement parameters (Å^2^)

**Table:**

	*x*	*y*	*z*	*U*_iso_*/*U*_eq_	Occ. (<1)
C1	0.8673 (3)	0.2938 (3)	0.8383 (5)	0.0409 (17)	
C2	0.6448 (4)	0.2228 (4)	0.8500 (6)	0.048 (2)	
C3	0.7169 (4)	0.3662 (5)	0.6559 (7)	0.061 (3)	
C4	0.7899 (5)	0.1428 (4)	0.6584 (7)	0.065 (3)	
C5	0.7782 (4)	0.4052 (3)	1.0092 (5)	0.0429 (19)	
C6	0.8231 (4)	0.3700 (4)	1.0184 (6)	0.051 (2)	
H6	0.8197	0.3332	1.0009	0.061*	
C7	0.8731 (4)	0.3883 (4)	1.0532 (7)	0.068 (3)	
H7	0.9033	0.3643	1.0575	0.081*	
C8	0.8776 (4)	0.4425 (4)	1.0812 (7)	0.066 (3)	
H8	0.9104	0.4548	1.1069	0.079*	
C9	0.8330 (4)	0.4789 (4)	1.0712 (6)	0.062 (2)	
H9	0.8365	0.5159	1.0882	0.074*	
C10	0.7845 (4)	0.4604 (3)	1.0366 (6)	0.047 (2)	
H10	0.7548	0.4848	1.0309	0.056*	
C11	0.6863 (14)	0.3427 (13)	1.0519 (15)	0.051 (8)	0.33 (5)
C12	0.7139 (17)	0.3339 (14)	1.1310 (18)	0.069 (10)	0.33 (5)
H12	0.7490	0.3489	1.1399	0.082*	0.33 (5)
C13	0.689 (2)	0.3025 (19)	1.1968 (17)	0.091 (14)	0.33 (5)
H13	0.7073	0.2966	1.2498	0.109*	0.33 (5)
C14	0.636 (2)	0.2801 (14)	1.1835 (17)	0.082 (12)	0.33 (5)
H14	0.6197	0.2591	1.2275	0.098*	0.33 (5)
C15	0.6089 (13)	0.2889 (13)	1.104 (2)	0.061 (10)	0.33 (5)
H15	0.5738	0.2739	1.0954	0.073*	0.33 (5)
C16	0.6339 (14)	0.3202 (17)	1.0385 (19)	0.047 (8)	0.33 (5)
H16	0.6155	0.3262	0.9856	0.056*	0.33 (5)
C11'	0.6779 (6)	0.3423 (6)	1.0508 (6)	0.037 (4)	0.67 (5)
C12'	0.6908 (9)	0.3538 (9)	1.1381 (7)	0.071 (7)	0.67 (5)
H12'	0.7205	0.3769	1.1515	0.085*	0.67 (5)
C13'	0.6592 (11)	0.3306 (12)	1.2053 (6)	0.091 (9)	0.67 (5)
H13'	0.6678	0.3383	1.2636	0.109*	0.67 (5)
C14'	0.6148 (8)	0.2960 (8)	1.1852 (8)	0.077 (6)	0.67 (5)
H14'	0.5937	0.2805	1.2301	0.093*	0.67 (5)
C15'	0.6020 (5)	0.2845 (6)	1.0979 (9)	0.055 (5)	0.67 (5)
H15'	0.5723	0.2613	1.0844	0.066*	0.67 (5)
C16'	0.6336 (6)	0.3076 (7)	1.0307 (7)	0.033 (3)	0.67 (5)
H16'	0.6250	0.2999	0.9723	0.039*	0.67 (5)
C17	0.6705 (4)	0.4354 (3)	0.9358 (6)	0.0424 (19)	
C18	0.6422 (4)	0.4652 (4)	1.0020 (7)	0.047 (2)	
H18	0.6478	0.4570	1.0611	0.056*	
C19	0.6053 (4)	0.5077 (4)	0.9767 (8)	0.055 (3)	
H19	0.5858	0.5270	1.0197	0.066*	
C20	0.5976 (5)	0.5213 (5)	0.8902 (8)	0.070 (3)	
H20	0.5728	0.5493	0.8750	0.085*	
C21	0.6264 (6)	0.4937 (5)	0.8258 (9)	0.082 (4)	
H21	0.6219	0.5036	0.7671	0.098*	
C22	0.6627 (5)	0.4504 (4)	0.8490 (7)	0.059 (3)	
H22	0.6818	0.4314	0.8050	0.071*	
C23	0.7351 (4)	0.1150 (3)	1.0213 (5)	0.0449 (19)	
C24	0.7267 (4)	0.0618 (3)	1.0496 (5)	0.045 (2)	
H24	0.7537	0.0347	1.0404	0.054*	
C25	0.6774 (4)	0.0486 (4)	1.0922 (6)	0.059 (2)	
H25	0.6715	0.0123	1.1110	0.071*	
C26	0.6381 (4)	0.0873 (4)	1.1067 (6)	0.069 (3)	
H26	0.6055	0.0777	1.1358	0.083*	
C27	0.6461 (5)	0.1411 (4)	1.0785 (6)	0.077 (3)	
H27	0.6192	0.1680	1.0889	0.092*	
C28	0.6937 (4)	0.1545 (3)	1.0353 (6)	0.061 (3)	
H28	0.6987	0.1906	1.0148	0.073*	
C29	0.8381 (4)	0.1725 (3)	1.0490 (6)	0.049 (2)	
C30	0.8807 (4)	0.2079 (3)	1.0226 (6)	0.0452 (19)	
H30	0.8876	0.2128	0.9629	0.054*	
C31	0.9129 (4)	0.2356 (4)	1.0827 (6)	0.053 (2)	
H31	0.9411	0.2591	1.0638	0.063*	
C32	0.9033 (5)	0.2286 (4)	1.1700 (6)	0.077 (3)	
H32	0.9255	0.2467	1.2111	0.093*	
C33	0.8608 (6)	0.1950 (7)	1.1974 (7)	0.118 (6)	
H33	0.8530	0.1916	1.2571	0.141*	
C34	0.8299 (5)	0.1662 (5)	1.1369 (6)	0.082 (3)	
H34	0.8027	0.1417	1.1565	0.099*	
C35	0.8372 (4)	0.0753 (3)	0.9380 (6)	0.044 (2)	
C36	0.8414 (4)	0.0592 (4)	0.8511 (7)	0.055 (2)	
H36	0.8241	0.0797	0.8072	0.066*	
C37	0.8715 (5)	0.0124 (4)	0.8297 (8)	0.069 (3)	
H37	0.8738	0.0014	0.7712	0.083*	
C38	0.8979 (5)	−0.0180 (4)	0.8922 (8)	0.061 (3)	
H38	0.9192	−0.0487	0.8767	0.073*	
C39	0.8925 (4)	−0.0025 (4)	0.9785 (7)	0.049 (2)	
H39	0.9098	−0.0233	1.0221	0.058*	
C40	0.8621 (4)	0.0431 (4)	1.0013 (7)	0.046 (2)	
H40	0.8582	0.0525	1.0603	0.055*	
C41	0.9331 (4)	0.3342 (4)	0.5584 (5)	0.0397 (19)	
C42	0.9634 (4)	0.3624 (4)	0.4946 (6)	0.044 (2)	
H42	0.9531	0.3595	0.4358	0.053*	
C43	1.0084 (4)	0.3948 (4)	0.5177 (6)	0.044 (2)	
H43	1.0276	0.4141	0.4745	0.052*	
C44	1.0250 (4)	0.3986 (4)	0.6035 (6)	0.050 (2)	
H44	1.0551	0.4208	0.6188	0.060*	
C45	0.9962 (3)	0.3688 (4)	0.6677 (6)	0.053 (2)	
H45	1.0077	0.3700	0.7260	0.064*	
C46	0.9502 (4)	0.3371 (4)	0.6440 (6)	0.049 (2)	
H46	0.9308	0.3177	0.6870	0.058*	
C47	0.9041 (9)	0.2240 (7)	0.4851 (14)	0.040 (4)	0.54 (4)
C48	0.8678 (8)	0.1797 (8)	0.4749 (13)	0.048 (5)	0.54 (4)
H48	0.8306	0.1836	0.4904	0.058*	0.54 (4)
C49	0.8870 (10)	0.1294 (7)	0.4417 (14)	0.058 (6)	0.54 (4)
H49	0.8626	0.0998	0.4349	0.070*	0.54 (4)
C50	0.9425 (11)	0.1236 (7)	0.4186 (15)	0.063 (6)	0.54 (4)
H50	0.9553	0.0900	0.3964	0.076*	0.54 (4)
C51	0.9788 (9)	0.1679 (8)	0.4288 (17)	0.074 (8)	0.54 (4)
H51	1.0160	0.1640	0.4133	0.089*	0.54 (4)
C52	0.9597 (9)	0.2182 (7)	0.4620 (17)	0.060 (7)	0.54 (4)
H52	0.9840	0.2479	0.4688	0.073*	0.54 (4)
C53	0.8431 (9)	0.3244 (9)	0.4421 (9)	0.043 (4)	0.54 (4)
C54	0.8090 (10)	0.3693 (8)	0.4617 (9)	0.049 (5)	0.54 (4)
H54	0.8013	0.3781	0.5200	0.059*	0.54 (4)
C55	0.7865 (9)	0.4011 (7)	0.3943 (11)	0.050 (5)	0.54 (4)
H55	0.7637	0.4312	0.4074	0.061*	0.54 (4)
C56	0.7981 (10)	0.3880 (9)	0.3071 (10)	0.056 (6)	0.54 (4)
H56	0.7830	0.4092	0.2620	0.067*	0.54 (4)
C57	0.8321 (9)	0.3430 (10)	0.2875 (8)	0.071 (7)	0.54 (4)
H57	0.8398	0.3342	0.2292	0.085*	0.54 (4)
C58	0.8546 (8)	0.3113 (10)	0.3550 (10)	0.061 (6)	0.54 (4)
H58	0.8774	0.2812	0.3418	0.073*	0.54 (4)
C47'	0.8901 (10)	0.2357 (9)	0.4762 (16)	0.042 (5)	0.46 (4)
C48'	0.8503 (10)	0.1945 (11)	0.4651 (17)	0.057 (7)	0.46 (4)
H48'	0.8141	0.2004	0.4843	0.069*	0.46 (4)
C49'	0.8645 (11)	0.1445 (10)	0.4255 (18)	0.065 (8)	0.46 (4)
H49'	0.8379	0.1169	0.4181	0.078*	0.46 (4)
C50'	0.9186 (12)	0.1357 (9)	0.3968 (17)	0.064 (8)	0.46 (4)
H50'	0.9282	0.1022	0.3703	0.077*	0.46 (4)
C51'	0.9584 (10)	0.1768 (9)	0.4079 (17)	0.058 (7)	0.46 (4)
H51'	0.9946	0.1709	0.3887	0.070*	0.46 (4)
C52'	0.9442 (10)	0.2268 (8)	0.4476 (17)	0.047 (6)	0.46 (4)
H52'	0.9708	0.2544	0.4550	0.056*	0.46 (4)
C53'	0.8337 (10)	0.3409 (11)	0.4448 (10)	0.038 (5)	0.46 (4)
C54'	0.7969 (11)	0.3816 (11)	0.4729 (10)	0.051 (6)	0.46 (4)
H54'	0.7908	0.3871	0.5327	0.061*	0.46 (4)
C55'	0.7692 (11)	0.4143 (10)	0.4116 (13)	0.050 (6)	0.46 (4)
H55'	0.7446	0.4416	0.4303	0.060*	0.46 (4)
C56'	0.7783 (11)	0.4062 (11)	0.3222 (12)	0.058 (6)	0.46 (4)
H56'	0.7598	0.4281	0.2812	0.070*	0.46 (4)
C57'	0.8151 (11)	0.3654 (12)	0.2942 (9)	0.068 (8)	0.46 (4)
H57'	0.8213	0.3600	0.2344	0.081*	0.46 (4)
C58'	0.8428 (9)	0.3328 (12)	0.3555 (11)	0.057 (6)	0.46 (4)
H58'	0.8675	0.3055	0.3367	0.068*	0.46 (4)
C59	0.5735 (4)	0.1679 (5)	0.5686 (7)	0.056 (2)	
C60	0.5604 (4)	0.1575 (5)	0.6566 (7)	0.065 (3)	
H60	0.5815	0.1739	0.7008	0.079*	
C61	0.5168 (4)	0.1233 (5)	0.6788 (8)	0.074 (3)	
H61	0.5079	0.1171	0.7375	0.089*	
C62	0.4865 (4)	0.0984 (5)	0.6125 (9)	0.070 (3)	
H62	0.4575	0.0745	0.6267	0.083*	
C63	0.4990 (4)	0.1087 (4)	0.5248 (8)	0.057 (3)	
H63	0.4780	0.0923	0.4806	0.069*	
C64	0.5421 (4)	0.1430 (4)	0.5033 (8)	0.054 (2)	
H64	0.5503	0.1497	0.4445	0.064*	
C65	0.5990 (8)	0.2824 (7)	0.4929 (13)	0.060 (5)	0.55 (2)
C66	0.5440 (8)	0.2869 (7)	0.4663 (15)	0.075 (7)	0.55 (2)
H66	0.5201	0.2568	0.4723	0.090*	0.55 (2)
C67	0.5246 (8)	0.3364 (9)	0.4307 (15)	0.099 (9)	0.55 (2)
H67	0.4878	0.3394	0.4129	0.118*	0.55 (2)
C68	0.5603 (10)	0.3814 (8)	0.4218 (14)	0.098 (9)	0.55 (2)
H68	0.5474	0.4146	0.3980	0.117*	0.55 (2)
C69	0.6153 (9)	0.3769 (7)	0.4484 (14)	0.090 (8)	0.55 (2)
H69	0.6392	0.4070	0.4424	0.107*	0.55 (2)
C70	0.6347 (7)	0.3274 (8)	0.4840 (13)	0.077 (7)	0.55 (2)
H70	0.6715	0.3244	0.5018	0.093*	0.55 (2)
C71	0.6663 (6)	0.1856 (7)	0.4556 (8)	0.055 (4)	0.55 (2)
C72	0.6701 (7)	0.2088 (9)	0.3721 (9)	0.086 (7)	0.55 (2)
H72	0.6537	0.2431	0.3607	0.103*	0.55 (2)
C73	0.6984 (8)	0.1809 (10)	0.3058 (8)	0.132 (12)	0.55 (2)
H73	0.7010	0.1965	0.2500	0.158*	0.55 (2)
C74	0.7229 (8)	0.1298 (9)	0.3230 (9)	0.090 (7)	0.55 (2)
H74	0.7418	0.1111	0.2787	0.109*	0.55 (2)
C75	0.7190 (7)	0.1066 (7)	0.4065 (11)	0.058 (5)	0.55 (2)
H75	0.7354	0.0724	0.4180	0.070*	0.55 (2)
C76	0.6907 (7)	0.1345 (7)	0.4728 (8)	0.054 (5)	0.55 (2)
H76	0.6882	0.1189	0.5286	0.065*	0.55 (2)
C65'	0.6148 (8)	0.2653 (8)	0.4851 (14)	0.052 (5)	0.45 (2)
C66'	0.5606 (7)	0.2743 (7)	0.4572 (14)	0.045 (5)	0.45 (2)
H66'	0.5333	0.2481	0.4689	0.054*	0.45 (2)
C67'	0.5473 (7)	0.3226 (8)	0.4117 (13)	0.059 (6)	0.45 (2)
H67'	0.5110	0.3287	0.3930	0.070*	0.45 (2)
C68'	0.5882 (9)	0.3618 (8)	0.3942 (13)	0.079 (8)	0.45 (2)
H68'	0.5792	0.3941	0.3638	0.095*	0.45 (2)
C69'	0.6424 (9)	0.3528 (10)	0.4221 (14)	0.075 (8)	0.45 (2)
H69'	0.6697	0.3790	0.4104	0.090*	0.45 (2)
C70'	0.6557 (7)	0.3045 (10)	0.4675 (14)	0.085 (9)	0.45 (2)
H70'	0.6920	0.2984	0.4862	0.101*	0.45 (2)
C71'	0.6754 (7)	0.1621 (9)	0.4575 (9)	0.047 (5)	0.45 (2)
C72'	0.6701 (7)	0.1719 (11)	0.3677 (10)	0.085 (9)	0.45 (2)
H72'	0.6465	0.1997	0.3476	0.102*	0.45 (2)
C73'	0.7001 (8)	0.1401 (13)	0.3081 (8)	0.088 (10)	0.45 (2)
H73'	0.6966	0.1466	0.2480	0.106*	0.45 (2)
C74'	0.7354 (7)	0.0985 (10)	0.3381 (9)	0.067 (6)	0.45 (2)
H74'	0.7555	0.0773	0.2982	0.080*	0.45 (2)
C75'	0.7407 (8)	0.0888 (7)	0.4279 (10)	0.054 (5)	0.45 (2)
H75'	0.7642	0.0610	0.4480	0.065*	0.45 (2)
C76'	0.7106 (8)	0.1206 (8)	0.4875 (8)	0.043 (5)	0.45 (2)
H76'	0.7142	0.1141	0.5476	0.051*	0.45 (2)
N1	0.8257 (3)	0.2813 (3)	0.8112 (4)	0.0341 (14)	
N2	0.6857 (3)	0.2327 (3)	0.8197 (4)	0.0393 (15)	
N3	0.7290 (3)	0.3253 (3)	0.6838 (5)	0.0459 (17)	
N4	0.7788 (3)	0.1844 (3)	0.6855 (5)	0.0451 (17)	
O1	0.9122 (3)	0.3084 (3)	0.8681 (4)	0.0664 (19)	
O2	0.5992 (3)	0.2124 (4)	0.8821 (5)	0.092 (3)	
O3	0.7042 (5)	0.4113 (4)	0.6237 (6)	0.103 (3)	
O4	0.8006 (5)	0.0971 (3)	0.6279 (6)	0.115 (4)	
P1	0.71594 (10)	0.37703 (9)	0.96044 (15)	0.0383 (5)	
P2	0.79767 (11)	0.13798 (9)	0.96362 (15)	0.0415 (6)	
Ag1	0.73407 (3)	0.31900 (2)	0.83917 (5)	0.03668 (17)	
Ag2	0.77858 (3)	0.19423 (2)	0.84006 (5)	0.03938 (19)	
Ag3	0.8158 (2)	0.2743 (2)	0.6536 (4)	0.0437 (11)	0.54 (4)
P3	0.8762 (8)	0.2884 (8)	0.5344 (13)	0.040 (3)	0.54 (4)
P4	0.6275 (5)	0.2197 (6)	0.5440 (9)	0.053 (2)	0.55 (2)
Ag4	0.6860 (3)	0.2386 (3)	0.6666 (5)	0.0439 (9)	0.55 (2)
P3'	0.8671 (9)	0.2982 (10)	0.5300 (14)	0.037 (3)	0.46 (4)
Ag3'	0.8134 (2)	0.2778 (3)	0.6582 (5)	0.0420 (12)	0.46 (4)
P4'	0.6390 (6)	0.2041 (7)	0.5432 (10)	0.045 (2)	0.45 (2)
Ag4'	0.6947 (3)	0.2250 (4)	0.6646 (6)	0.0417 (11)	0.45 (2)

### Atomic displacement parameters (Å^2^)

**Table:**

	*U*^11^	*U*^22^	*U*^33^	*U*^12^	*U*^13^	*U*^23^
C1	0.048 (4)	0.049 (4)	0.026 (4)	−0.005 (3)	0.006 (3)	0.002 (3)
C2	0.059 (4)	0.061 (5)	0.025 (4)	−0.023 (4)	0.001 (3)	−0.004 (4)
C3	0.072 (6)	0.070 (5)	0.041 (5)	−0.002 (4)	0.009 (5)	0.014 (4)
C4	0.100 (7)	0.055 (4)	0.040 (5)	−0.023 (4)	0.011 (5)	−0.005 (4)
C5	0.059 (4)	0.032 (4)	0.037 (4)	0.001 (3)	0.006 (3)	−0.002 (3)
C6	0.064 (5)	0.041 (4)	0.047 (5)	0.008 (3)	−0.013 (4)	−0.011 (4)
C7	0.071 (5)	0.052 (5)	0.080 (7)	0.017 (4)	−0.016 (5)	−0.026 (5)
C8	0.058 (5)	0.056 (5)	0.083 (7)	0.005 (4)	−0.013 (5)	−0.021 (5)
C9	0.065 (5)	0.040 (5)	0.079 (7)	0.002 (4)	0.001 (4)	−0.015 (4)
C10	0.056 (5)	0.031 (4)	0.052 (5)	0.002 (3)	0.004 (4)	−0.003 (3)
C11	0.075 (12)	0.040 (15)	0.037 (9)	−0.005 (10)	0.002 (8)	0.000 (9)
C12	0.093 (16)	0.07 (2)	0.041 (10)	−0.005 (15)	−0.006 (11)	0.009 (11)
C13	0.11 (2)	0.11 (3)	0.057 (13)	−0.02 (2)	−0.003 (13)	0.026 (16)
C14	0.10 (2)	0.08 (2)	0.059 (14)	−0.005 (17)	0.007 (12)	0.010 (13)
C15	0.091 (18)	0.036 (18)	0.055 (14)	−0.005 (15)	0.011 (11)	−0.002 (11)
C16	0.069 (12)	0.027 (16)	0.045 (12)	0.008 (10)	0.008 (9)	−0.005 (9)
C11'	0.042 (7)	0.037 (8)	0.033 (6)	−0.008 (6)	−0.001 (4)	0.004 (5)
C12'	0.078 (12)	0.100 (14)	0.035 (6)	−0.039 (11)	−0.003 (6)	0.007 (6)
C13'	0.102 (16)	0.135 (19)	0.036 (7)	−0.054 (15)	−0.001 (7)	0.021 (7)
C14'	0.070 (12)	0.101 (14)	0.061 (8)	−0.026 (10)	−0.007 (6)	0.028 (7)
C15'	0.036 (7)	0.067 (13)	0.063 (8)	−0.003 (8)	−0.001 (6)	0.022 (7)
C16'	0.032 (6)	0.025 (7)	0.042 (7)	0.009 (5)	−0.002 (5)	−0.002 (5)
C17	0.049 (4)	0.031 (4)	0.048 (4)	−0.005 (3)	0.007 (3)	0.003 (3)
C18	0.043 (5)	0.040 (4)	0.057 (5)	−0.008 (3)	0.011 (4)	−0.001 (4)
C19	0.040 (5)	0.048 (5)	0.076 (6)	−0.003 (4)	0.011 (4)	−0.006 (4)
C20	0.082 (8)	0.052 (6)	0.077 (6)	0.016 (5)	−0.006 (5)	0.002 (5)
C21	0.120 (9)	0.059 (6)	0.067 (7)	0.035 (6)	−0.009 (6)	0.008 (5)
C22	0.089 (7)	0.039 (4)	0.049 (5)	0.017 (4)	0.008 (5)	0.005 (4)
C23	0.078 (5)	0.030 (4)	0.026 (4)	0.005 (3)	0.005 (4)	0.007 (3)
C24	0.065 (5)	0.031 (4)	0.040 (4)	0.008 (3)	0.003 (4)	0.009 (3)
C25	0.077 (6)	0.040 (4)	0.059 (6)	0.003 (4)	0.012 (4)	0.017 (4)
C26	0.089 (6)	0.058 (5)	0.061 (6)	0.024 (5)	0.033 (5)	0.025 (5)
C27	0.107 (7)	0.057 (5)	0.067 (7)	0.029 (5)	0.051 (6)	0.028 (5)
C28	0.101 (6)	0.032 (4)	0.049 (5)	0.014 (4)	0.032 (5)	0.012 (4)
C29	0.071 (5)	0.033 (4)	0.043 (4)	−0.004 (4)	−0.001 (4)	−0.010 (3)
C30	0.059 (5)	0.032 (4)	0.044 (4)	0.011 (3)	−0.002 (3)	−0.005 (3)
C31	0.054 (5)	0.049 (5)	0.055 (5)	0.004 (4)	0.000 (4)	−0.019 (4)
C32	0.103 (8)	0.079 (7)	0.050 (5)	−0.015 (5)	−0.003 (5)	−0.024 (5)
C33	0.157 (12)	0.157 (12)	0.040 (6)	−0.074 (10)	0.016 (6)	−0.035 (6)
C34	0.122 (9)	0.090 (7)	0.035 (4)	−0.044 (6)	0.007 (5)	−0.013 (4)
C35	0.055 (5)	0.032 (4)	0.045 (4)	−0.006 (3)	−0.004 (3)	−0.007 (3)
C36	0.087 (6)	0.042 (4)	0.037 (4)	0.004 (4)	−0.006 (4)	−0.005 (4)
C37	0.099 (7)	0.055 (5)	0.054 (6)	0.016 (5)	−0.002 (5)	−0.009 (4)
C38	0.073 (7)	0.040 (5)	0.068 (6)	0.005 (4)	−0.001 (5)	−0.006 (4)
C39	0.052 (5)	0.041 (5)	0.053 (5)	−0.003 (4)	−0.002 (4)	−0.003 (4)
C40	0.048 (5)	0.039 (4)	0.050 (5)	−0.006 (3)	−0.008 (4)	−0.002 (3)
C41	0.045 (4)	0.046 (4)	0.029 (4)	−0.008 (3)	0.010 (3)	−0.002 (3)
C42	0.051 (5)	0.043 (5)	0.038 (4)	−0.005 (4)	0.006 (3)	0.003 (3)
C43	0.041 (4)	0.044 (5)	0.045 (4)	−0.007 (3)	0.007 (4)	0.003 (4)
C44	0.029 (4)	0.066 (6)	0.054 (5)	−0.004 (4)	0.004 (3)	−0.011 (4)
C45	0.043 (4)	0.075 (5)	0.041 (4)	−0.002 (4)	0.004 (4)	−0.016 (4)
C46	0.048 (4)	0.068 (5)	0.029 (4)	−0.011 (4)	0.006 (3)	−0.003 (4)
C47	0.048 (9)	0.047 (7)	0.024 (8)	−0.015 (6)	0.003 (7)	0.001 (6)
C48	0.054 (10)	0.038 (8)	0.052 (11)	−0.013 (7)	0.012 (8)	−0.008 (7)
C49	0.069 (12)	0.048 (9)	0.058 (13)	−0.009 (7)	0.010 (10)	−0.019 (8)
C50	0.070 (12)	0.052 (9)	0.067 (15)	−0.002 (8)	0.016 (10)	−0.014 (9)
C51	0.070 (12)	0.068 (10)	0.085 (19)	−0.010 (8)	0.020 (11)	−0.017 (10)
C52	0.057 (10)	0.062 (10)	0.062 (16)	−0.012 (7)	0.018 (9)	−0.007 (9)
C53	0.064 (10)	0.040 (9)	0.026 (6)	−0.012 (7)	0.002 (5)	0.002 (5)
C54	0.074 (13)	0.046 (9)	0.028 (8)	−0.008 (8)	0.007 (7)	0.004 (6)
C55	0.066 (12)	0.044 (10)	0.041 (8)	−0.015 (8)	−0.001 (8)	0.003 (7)
C56	0.072 (14)	0.069 (13)	0.028 (8)	0.008 (10)	−0.008 (7)	0.000 (7)
C57	0.098 (15)	0.091 (14)	0.023 (7)	0.032 (12)	−0.003 (7)	0.005 (7)
C58	0.081 (12)	0.077 (12)	0.025 (6)	0.023 (10)	−0.001 (6)	0.000 (7)
C47'	0.054 (10)	0.052 (8)	0.021 (9)	−0.009 (6)	0.013 (8)	0.002 (7)
C48'	0.058 (12)	0.057 (11)	0.057 (15)	−0.008 (8)	0.013 (10)	−0.011 (10)
C49'	0.062 (13)	0.060 (11)	0.072 (18)	−0.009 (9)	0.018 (11)	−0.014 (11)
C50'	0.061 (13)	0.056 (11)	0.075 (19)	−0.009 (9)	0.016 (11)	−0.022 (11)
C51'	0.059 (12)	0.060 (10)	0.055 (15)	−0.012 (8)	0.034 (11)	−0.020 (9)
C52'	0.054 (11)	0.053 (10)	0.034 (11)	−0.011 (7)	0.014 (9)	−0.010 (8)
C53'	0.044 (10)	0.048 (11)	0.022 (7)	−0.016 (8)	0.000 (6)	0.003 (7)
C54'	0.055 (12)	0.059 (12)	0.038 (9)	−0.013 (9)	−0.006 (7)	−0.003 (7)
C55'	0.055 (13)	0.058 (12)	0.038 (9)	−0.012 (9)	−0.006 (8)	−0.001 (8)
C56'	0.065 (14)	0.075 (14)	0.035 (9)	−0.002 (10)	−0.003 (8)	−0.008 (8)
C57'	0.099 (17)	0.089 (17)	0.016 (8)	0.025 (13)	−0.009 (8)	−0.003 (8)
C58'	0.064 (12)	0.080 (14)	0.026 (7)	0.003 (10)	0.001 (7)	0.005 (8)
C59	0.040 (4)	0.075 (6)	0.052 (5)	−0.010 (4)	−0.005 (3)	−0.012 (4)
C60	0.044 (4)	0.099 (7)	0.054 (5)	−0.013 (4)	0.001 (4)	−0.001 (5)
C61	0.044 (5)	0.108 (8)	0.070 (6)	−0.008 (5)	−0.001 (4)	0.008 (5)
C62	0.039 (5)	0.076 (7)	0.094 (7)	0.007 (4)	−0.002 (4)	0.002 (5)
C63	0.036 (4)	0.058 (6)	0.077 (6)	0.007 (4)	−0.013 (4)	−0.008 (5)
C64	0.041 (4)	0.055 (5)	0.065 (6)	0.003 (4)	−0.009 (4)	−0.021 (4)
C65	0.065 (10)	0.077 (9)	0.038 (9)	−0.007 (6)	−0.007 (8)	−0.006 (7)
C66	0.068 (11)	0.088 (12)	0.067 (15)	−0.001 (8)	−0.015 (10)	−0.016 (10)
C67	0.095 (14)	0.099 (13)	0.10 (2)	0.002 (9)	−0.023 (13)	−0.002 (13)
C68	0.101 (14)	0.101 (13)	0.09 (2)	−0.002 (10)	−0.034 (13)	0.014 (12)
C69	0.096 (14)	0.086 (11)	0.087 (18)	−0.008 (9)	−0.033 (12)	0.021 (11)
C70	0.074 (12)	0.087 (10)	0.070 (14)	−0.016 (8)	−0.033 (10)	0.019 (10)
C71	0.042 (8)	0.085 (11)	0.039 (6)	−0.016 (7)	−0.002 (6)	−0.022 (6)
C72	0.108 (16)	0.116 (14)	0.034 (7)	0.010 (12)	0.008 (7)	−0.012 (8)
C73	0.19 (3)	0.153 (18)	0.055 (10)	0.055 (19)	0.040 (12)	0.001 (11)
C74	0.096 (17)	0.126 (16)	0.049 (9)	0.006 (13)	0.022 (9)	−0.016 (9)
C75	0.040 (10)	0.087 (12)	0.047 (9)	−0.028 (8)	0.005 (7)	−0.029 (8)
C76	0.043 (11)	0.082 (11)	0.037 (8)	−0.020 (8)	0.001 (7)	−0.017 (7)
C65'	0.044 (9)	0.079 (10)	0.034 (10)	−0.013 (7)	−0.010 (7)	−0.005 (8)
C66'	0.040 (9)	0.064 (10)	0.031 (10)	−0.013 (7)	−0.011 (8)	−0.006 (8)
C67'	0.066 (12)	0.069 (11)	0.041 (11)	−0.012 (8)	−0.017 (9)	−0.008 (8)
C68'	0.083 (13)	0.092 (14)	0.063 (16)	−0.031 (10)	−0.028 (11)	0.021 (12)
C69'	0.080 (13)	0.103 (14)	0.042 (13)	−0.032 (10)	−0.025 (10)	0.020 (12)
C70'	0.065 (11)	0.110 (14)	0.08 (2)	−0.036 (10)	−0.029 (11)	0.033 (14)
C71'	0.035 (9)	0.073 (12)	0.031 (7)	−0.015 (8)	−0.006 (6)	−0.009 (7)
C72'	0.088 (16)	0.14 (2)	0.029 (8)	0.032 (16)	−0.004 (7)	0.000 (8)
C73'	0.091 (18)	0.14 (2)	0.035 (9)	0.024 (15)	0.003 (9)	−0.002 (9)
C74'	0.051 (11)	0.106 (16)	0.044 (9)	−0.011 (10)	0.002 (7)	−0.003 (8)
C75'	0.049 (11)	0.076 (12)	0.038 (8)	−0.014 (9)	−0.005 (7)	−0.006 (7)
C76'	0.032 (10)	0.065 (10)	0.032 (8)	−0.020 (7)	−0.009 (6)	−0.005 (7)
N1	0.048 (3)	0.033 (3)	0.021 (3)	−0.004 (3)	0.002 (2)	0.002 (2)
N2	0.053 (4)	0.037 (3)	0.028 (3)	−0.011 (3)	−0.002 (3)	−0.005 (2)
N3	0.048 (4)	0.059 (4)	0.031 (4)	−0.014 (3)	−0.002 (3)	0.000 (3)
N4	0.060 (4)	0.052 (4)	0.024 (3)	−0.019 (3)	0.003 (3)	0.001 (3)
O1	0.047 (3)	0.113 (5)	0.039 (3)	−0.029 (3)	−0.001 (3)	0.003 (3)
O2	0.064 (4)	0.148 (7)	0.063 (5)	−0.050 (4)	0.017 (4)	−0.006 (5)
O3	0.160 (9)	0.076 (5)	0.073 (6)	0.014 (5)	−0.005 (6)	0.026 (4)
O4	0.220 (11)	0.050 (4)	0.075 (6)	−0.008 (5)	0.038 (7)	−0.022 (4)
P1	0.0541 (13)	0.0287 (10)	0.0320 (11)	−0.0015 (9)	0.0029 (9)	0.0003 (9)
P2	0.0661 (15)	0.0279 (10)	0.0304 (11)	−0.0005 (10)	0.0016 (10)	−0.0020 (9)
Ag1	0.0482 (4)	0.0319 (3)	0.0299 (4)	−0.0017 (2)	−0.0001 (4)	−0.0009 (3)
Ag2	0.0587 (4)	0.0303 (3)	0.0291 (4)	−0.0039 (2)	0.0038 (3)	0.0004 (3)
Ag3	0.070 (3)	0.0413 (17)	0.0194 (13)	−0.0175 (12)	0.0076 (12)	0.0076 (12)
P3	0.049 (6)	0.045 (5)	0.025 (3)	−0.014 (4)	0.001 (3)	0.006 (3)
P4	0.043 (4)	0.077 (6)	0.038 (3)	−0.008 (3)	−0.004 (3)	−0.010 (4)
Ag4	0.0490 (16)	0.056 (2)	0.0271 (11)	−0.0101 (13)	−0.0026 (11)	−0.0058 (15)
P3'	0.039 (5)	0.052 (7)	0.019 (4)	−0.011 (4)	0.009 (3)	0.000 (4)
Ag3'	0.027 (2)	0.070 (3)	0.0293 (18)	−0.0199 (13)	0.0096 (13)	−0.0093 (18)
P4'	0.037 (5)	0.071 (6)	0.027 (3)	−0.009 (4)	−0.009 (3)	−0.013 (4)
Ag4'	0.0395 (18)	0.057 (3)	0.0284 (11)	−0.0175 (16)	−0.0047 (14)	−0.0126 (18)

### Geometric parameters (Å, º)

**Table:**

C1—N1	1.123 (10)	C54—H54	0.9300
C1—O1	1.225 (10)	C55—C56	1.3900
C2—N2	1.114 (10)	C55—H55	0.9300
C2—O2	1.227 (10)	C56—C57	1.3900
C3—N3	1.111 (12)	C56—H56	0.9300
C3—O3	1.231 (13)	C57—C58	1.3900
C4—N4	1.115 (12)	C57—H57	0.9300
C4—O4	1.222 (12)	C58—H58	0.9300
C5—C6	1.380 (11)	C47'—C48'	1.3900
C5—C10	1.401 (10)	C47'—C52'	1.3900
C5—P1	1.805 (9)	C47'—P3'	1.80 (2)
C6—C7	1.389 (12)	C48'—C49'	1.3900
C6—H6	0.9300	C48'—H48'	0.9300
C7—C8	1.376 (12)	C49'—C50'	1.3900
C7—H7	0.9300	C49'—H49'	0.9300
C8—C9	1.395 (12)	C50'—C51'	1.3900
C8—H8	0.9300	C50'—H50'	0.9300
C9—C10	1.357 (12)	C51'—C52'	1.3900
C9—H9	0.9300	C51'—H51'	0.9300
C10—H10	0.9300	C52'—H52'	0.9300
C11—C12	1.3900	C53'—C54'	1.3900
C11—C16	1.3900	C53'—C58'	1.3900
C11—P1	1.767 (17)	C53'—P3'	1.84 (2)
C12—C13	1.3900	C54'—C55'	1.3900
C12—H12	0.9300	C54'—H54'	0.9300
C13—C14	1.3900	C55'—C56'	1.3900
C13—H13	0.9300	C55'—H55'	0.9300
C14—C15	1.3900	C56'—C57'	1.3900
C14—H14	0.9300	C56'—H56'	0.9300
C15—C16	1.3900	C57'—C58'	1.3900
C15—H15	0.9300	C57'—H57'	0.9300
C16—H16	0.9300	C58'—H58'	0.9300
C11'—C12'	1.3900	C59—C64	1.384 (12)
C11'—C16'	1.3900	C59—C60	1.397 (14)
C11'—P1	1.851 (9)	C59—P4	1.841 (17)
C12'—C13'	1.3900	C59—P4'	1.843 (18)
C12'—H12'	0.9300	C60—C61	1.377 (14)
C13'—C14'	1.3900	C60—H60	0.9300
C13'—H13'	0.9300	C61—C62	1.382 (15)
C14'—C15'	1.3900	C61—H61	0.9300
C14'—H14'	0.9300	C62—C63	1.390 (15)
C15'—C16'	1.3900	C62—H62	0.9300
C15'—H15'	0.9300	C63—C64	1.366 (14)
C16'—H16'	0.9300	C63—H63	0.9300
C17—C22	1.382 (13)	C64—H64	0.9300
C17—C18	1.412 (12)	C65—C66	1.3900
C17—P1	1.820 (9)	C65—C70	1.3900
C18—C19	1.409 (14)	C65—P4	1.832 (16)
C18—H18	0.9300	C66—C67	1.3900
C19—C20	1.368 (15)	C66—H66	0.9300
C19—H19	0.9300	C67—C68	1.3900
C20—C21	1.370 (17)	C67—H67	0.9300
C20—H20	0.9300	C68—C69	1.3900
C21—C22	1.408 (13)	C68—H68	0.9300
C21—H21	0.9300	C69—C70	1.3900
C22—H22	0.9300	C69—H69	0.9300
C23—C24	1.367 (10)	C70—H70	0.9300
C23—C28	1.396 (12)	C71—C72	1.3900
C23—P2	1.829 (9)	C71—C76	1.3900
C24—C25	1.389 (12)	C71—P4	1.831 (14)
C24—H24	0.9300	C72—C73	1.3900
C25—C26	1.348 (12)	C72—H72	0.9300
C25—H25	0.9300	C73—C74	1.3900
C26—C27	1.380 (12)	C73—H73	0.9300
C26—H26	0.9300	C74—C75	1.3900
C27—C28	1.360 (13)	C74—H74	0.9300
C27—H27	0.9300	C75—C76	1.3900
C28—H28	0.9300	C75—H75	0.9300
C29—C34	1.359 (12)	C76—H76	0.9300
C29—C30	1.394 (12)	C65'—C66'	1.3900
C29—P2	1.822 (8)	C65'—C70'	1.3900
C30—C31	1.371 (11)	C65'—P4'	1.815 (17)
C30—H30	0.9300	C66'—C67'	1.3900
C31—C32	1.358 (12)	C66'—H66'	0.9300
C31—H31	0.9300	C67'—C68'	1.3900
C32—C33	1.370 (15)	C67'—H67'	0.9300
C32—H32	0.9300	C68'—C69'	1.3900
C33—C34	1.371 (14)	C68'—H68'	0.9300
C33—H33	0.9300	C69'—C70'	1.3900
C34—H34	0.9300	C69'—H69'	0.9300
C35—C40	1.373 (13)	C70'—H70'	0.9300
C35—C36	1.381 (12)	C71'—C72'	1.3900
C35—P2	1.826 (9)	C71'—C76'	1.3900
C36—C37	1.379 (13)	C71'—P4'	1.868 (17)
C36—H36	0.9300	C72'—C73'	1.3900
C37—C38	1.357 (15)	C72'—H72'	0.9300
C37—H37	0.9300	C73'—C74'	1.3900
C38—C39	1.371 (14)	C73'—H73'	0.9300
C38—H38	0.9300	C74'—C75'	1.3900
C39—C40	1.366 (13)	C74'—H74'	0.9300
C39—H39	0.9300	C75'—C76'	1.3900
C40—H40	0.9300	C75'—H75'	0.9300
C41—C46	1.367 (12)	C76'—H76'	0.9300
C41—C42	1.391 (11)	N1—Ag3'	2.346 (9)
C41—P3	1.80 (2)	N1—Ag3	2.414 (8)
C41—P3'	1.86 (2)	N1—Ag1	2.424 (7)
C42—C43	1.381 (12)	N1—Ag2	2.425 (6)
C42—H42	0.9300	N2—Ag4	2.332 (10)
C43—C44	1.367 (13)	N2—Ag4'	2.376 (11)
C43—H43	0.9300	N2—Ag1	2.401 (6)
C44—C45	1.398 (13)	N2—Ag2	2.440 (8)
C44—H44	0.9300	N3—Ag4	2.345 (10)
C45—C46	1.392 (12)	N3—Ag3'	2.366 (11)
C45—H45	0.9300	N3—Ag1	2.371 (7)
C46—H46	0.9300	N3—Ag3	2.469 (11)
C47—C48	1.3900	N3—Ag4'	2.570 (12)
C47—C52	1.3900	N4—Ag4'	2.273 (13)
C47—P3	1.848 (19)	N4—Ag2	2.361 (7)
C48—C49	1.3900	N4—Ag3	2.391 (8)
C48—H48	0.9300	N4—Ag3'	2.435 (9)
C49—C50	1.3900	N4—Ag4	2.605 (12)
C49—H49	0.9300	P1—Ag1	2.354 (2)
C50—C51	1.3900	P2—Ag2	2.361 (2)
C50—H50	0.9300	Ag1—Ag2	3.1906 (10)
C51—C52	1.3900	Ag3—P3	2.35 (2)
C51—H51	0.9300	Ag3—Ag4	3.250 (9)
C52—H52	0.9300	P4—Ag4	2.381 (14)
C53—C54	1.3900	P3'—Ag3'	2.39 (2)
C53—C58	1.3900	Ag3'—Ag4'	3.133 (8)
C53—P3	1.832 (18)	P4'—Ag4'	2.336 (15)
C54—C55	1.3900		
			
N1—C1—O1	178.9 (10)	C61—C60—C59	120.9 (11)
N2—C2—O2	178.8 (12)	C61—C60—H60	119.6
N3—C3—O3	178.7 (13)	C59—C60—H60	119.6
N4—C4—O4	178.4 (14)	C60—C61—C62	119.0 (11)
C6—C5—C10	117.8 (8)	C60—C61—H61	120.5
C6—C5—P1	117.5 (6)	C62—C61—H61	120.5
C10—C5—P1	124.7 (7)	C61—C62—C63	120.4 (11)
C5—C6—C7	121.6 (8)	C61—C62—H62	119.8
C5—C6—H6	119.2	C63—C62—H62	119.8
C7—C6—H6	119.2	C64—C63—C62	120.2 (11)
C8—C7—C6	119.1 (9)	C64—C63—H63	119.9
C8—C7—H7	120.5	C62—C63—H63	119.9
C6—C7—H7	120.5	C63—C64—C59	120.3 (11)
C7—C8—C9	120.2 (9)	C63—C64—H64	119.9
C7—C8—H8	119.9	C59—C64—H64	119.9
C9—C8—H8	119.9	C66—C65—C70	120.0
C10—C9—C8	119.8 (8)	C66—C65—P4	123.0 (10)
C10—C9—H9	120.1	C70—C65—P4	116.9 (10)
C8—C9—H9	120.1	C67—C66—C65	120.0
C9—C10—C5	121.4 (8)	C67—C66—H66	120.0
C9—C10—H10	119.3	C65—C66—H66	120.0
C5—C10—H10	119.3	C66—C67—C68	120.0
C12—C11—C16	120.0	C66—C67—H67	120.0
C12—C11—P1	124.1 (17)	C68—C67—H67	120.0
C16—C11—P1	115.7 (17)	C69—C68—C67	120.0
C13—C12—C11	120.0	C69—C68—H68	120.0
C13—C12—H12	120.0	C67—C68—H68	120.0
C11—C12—H12	120.0	C70—C69—C68	120.0
C14—C13—C12	120.0	C70—C69—H69	120.0
C14—C13—H13	120.0	C68—C69—H69	120.0
C12—C13—H13	120.0	C69—C70—C65	120.0
C13—C14—C15	120.0	C69—C70—H70	120.0
C13—C14—H14	120.0	C65—C70—H70	120.0
C15—C14—H14	120.0	C72—C71—C76	120.0
C16—C15—C14	120.0	C72—C71—P4	121.6 (8)
C16—C15—H15	120.0	C76—C71—P4	118.4 (8)
C14—C15—H15	120.0	C71—C72—C73	120.0
C15—C16—C11	120.0	C71—C72—H72	120.0
C15—C16—H16	120.0	C73—C72—H72	120.0
C11—C16—H16	120.0	C74—C73—C72	120.0
C12'—C11'—C16'	120.0	C74—C73—H73	120.0
C12'—C11'—P1	120.6 (7)	C72—C73—H73	120.0
C16'—C11'—P1	119.2 (7)	C75—C74—C73	120.0
C13'—C12'—C11'	120.0	C75—C74—H74	120.0
C13'—C12'—H12'	120.0	C73—C74—H74	120.0
C11'—C12'—H12'	120.0	C76—C75—C74	120.0
C12'—C13'—C14'	120.0	C76—C75—H75	120.0
C12'—C13'—H13'	120.0	C74—C75—H75	120.0
C14'—C13'—H13'	120.0	C75—C76—C71	120.0
C15'—C14'—C13'	120.0	C75—C76—H76	120.0
C15'—C14'—H14'	120.0	C71—C76—H76	120.0
C13'—C14'—H14'	120.0	C66'—C65'—C70'	120.0
C14'—C15'—C16'	120.0	C66'—C65'—P4'	125.2 (10)
C14'—C15'—H15'	120.0	C70'—C65'—P4'	114.7 (10)
C16'—C15'—H15'	120.0	C67'—C66'—C65'	120.0
C15'—C16'—C11'	120.0	C67'—C66'—H66'	120.0
C15'—C16'—H16'	120.0	C65'—C66'—H66'	120.0
C11'—C16'—H16'	120.0	C66'—C67'—C68'	120.0
C22—C17—C18	118.9 (9)	C66'—C67'—H67'	120.0
C22—C17—P1	118.7 (7)	C68'—C67'—H67'	120.0
C18—C17—P1	122.5 (7)	C69'—C68'—C67'	120.0
C19—C18—C17	118.7 (10)	C69'—C68'—H68'	120.0
C19—C18—H18	120.7	C67'—C68'—H68'	120.0
C17—C18—H18	120.7	C68'—C69'—C70'	120.0
C20—C19—C18	121.4 (11)	C68'—C69'—H69'	120.0
C20—C19—H19	119.3	C70'—C69'—H69'	120.0
C18—C19—H19	119.3	C69'—C70'—C65'	120.0
C19—C20—C21	120.2 (11)	C69'—C70'—H70'	120.0
C19—C20—H20	119.9	C65'—C70'—H70'	120.0
C21—C20—H20	119.9	C72'—C71'—C76'	120.0
C20—C21—C22	119.7 (12)	C72'—C71'—P4'	123.4 (9)
C20—C21—H21	120.1	C76'—C71'—P4'	116.5 (9)
C22—C21—H21	120.1	C73'—C72'—C71'	120.0
C17—C22—C21	121.1 (11)	C73'—C72'—H72'	120.0
C17—C22—H22	119.4	C71'—C72'—H72'	120.0
C21—C22—H22	119.4	C74'—C73'—C72'	120.0
C24—C23—C28	119.1 (8)	C74'—C73'—H73'	120.0
C24—C23—P2	123.8 (7)	C72'—C73'—H73'	120.0
C28—C23—P2	117.1 (6)	C75'—C74'—C73'	120.0
C23—C24—C25	119.2 (8)	C75'—C74'—H74'	120.0
C23—C24—H24	120.4	C73'—C74'—H74'	120.0
C25—C24—H24	120.4	C74'—C75'—C76'	120.0
C26—C25—C24	121.3 (8)	C74'—C75'—H75'	120.0
C26—C25—H25	119.3	C76'—C75'—H75'	120.0
C24—C25—H25	119.3	C75'—C76'—C71'	120.0
C25—C26—C27	120.1 (9)	C75'—C76'—H76'	120.0
C25—C26—H26	120.0	C71'—C76'—H76'	120.0
C27—C26—H26	120.0	C1—N1—Ag3'	119.1 (6)
C28—C27—C26	119.4 (9)	C1—N1—Ag3	118.1 (6)
C28—C27—H27	120.3	C1—N1—Ag1	130.2 (6)
C26—C27—H27	120.3	Ag3'—N1—Ag1	94.2 (3)
C27—C28—C23	120.9 (8)	Ag3—N1—Ag1	96.3 (3)
C27—C28—H28	119.5	C1—N1—Ag2	125.7 (6)
C23—C28—H28	119.5	Ag3'—N1—Ag2	95.1 (3)
C34—C29—C30	117.2 (8)	Ag3—N1—Ag2	94.2 (2)
C34—C29—P2	124.9 (7)	Ag1—N1—Ag2	82.3 (2)
C30—C29—P2	117.9 (7)	C2—N2—Ag4	115.3 (7)
C31—C30—C29	121.5 (8)	C2—N2—Ag4'	118.3 (6)
C31—C30—H30	119.3	C2—N2—Ag1	124.3 (6)
C29—C30—H30	119.3	Ag4—N2—Ag1	94.0 (3)
C32—C31—C30	119.7 (9)	Ag4'—N2—Ag1	98.4 (3)
C32—C31—H31	120.1	C2—N2—Ag2	132.7 (7)
C30—C31—H31	120.1	Ag4—N2—Ag2	98.5 (3)
C31—C32—C33	119.8 (10)	Ag4'—N2—Ag2	90.8 (3)
C31—C32—H32	120.1	Ag1—N2—Ag2	82.5 (2)
C33—C32—H32	120.1	C3—N3—Ag4	129.2 (8)
C32—C33—C34	120.0 (10)	C3—N3—Ag3'	126.2 (8)
C32—C33—H33	120.0	C3—N3—Ag1	116.7 (8)
C34—C33—H33	120.0	Ag4—N3—Ag1	94.4 (3)
C29—C34—C33	121.7 (10)	Ag3'—N3—Ag1	95.1 (3)
C29—C34—H34	119.2	C3—N3—Ag3	126.3 (7)
C33—C34—H34	119.2	Ag4—N3—Ag3	84.9 (4)
C40—C35—C36	118.6 (9)	Ag1—N3—Ag3	96.3 (3)
C40—C35—P2	123.0 (7)	C3—N3—Ag4'	134.9 (8)
C36—C35—P2	118.4 (8)	Ag3'—N3—Ag4'	78.7 (3)
C37—C36—C35	119.6 (10)	Ag1—N3—Ag4'	94.0 (3)
C37—C36—H36	120.2	C4—N4—Ag4'	123.1 (8)
C35—C36—H36	120.2	C4—N4—Ag2	117.3 (8)
C38—C37—C36	121.5 (11)	Ag4'—N4—Ag2	95.4 (4)
C38—C37—H37	119.3	C4—N4—Ag3	130.5 (8)
C36—C37—H37	119.3	Ag2—N4—Ag3	96.5 (3)
C37—C38—C39	118.6 (10)	C4—N4—Ag3'	133.3 (8)
C37—C38—H38	120.7	Ag4'—N4—Ag3'	83.4 (4)
C39—C38—H38	120.7	Ag2—N4—Ag3'	94.5 (3)
C40—C39—C38	120.8 (10)	C4—N4—Ag4	127.8 (8)
C40—C39—H39	119.6	Ag2—N4—Ag4	93.3 (3)
C38—C39—H39	119.6	Ag3—N4—Ag4	81.0 (3)
C39—C40—C35	120.7 (10)	C11—P1—C5	100.8 (12)
C39—C40—H40	119.6	C11—P1—C17	106.3 (11)
C35—C40—H40	119.6	C5—P1—C17	107.0 (4)
C46—C41—C42	118.7 (8)	C5—P1—C11'	106.0 (5)
C46—C41—P3	117.1 (9)	C17—P1—C11'	101.8 (6)
C42—C41—P3	123.9 (9)	C11—P1—Ag1	114.4 (11)
C46—C41—P3'	120.1 (9)	C5—P1—Ag1	113.0 (3)
C42—C41—P3'	120.9 (9)	C17—P1—Ag1	114.1 (3)
C43—C42—C41	120.7 (9)	C11'—P1—Ag1	113.9 (5)
C43—C42—H42	119.7	C29—P2—C35	104.5 (4)
C41—C42—H42	119.7	C29—P2—C23	103.6 (4)
C44—C43—C42	120.8 (9)	C35—P2—C23	106.4 (4)
C44—C43—H43	119.6	C29—P2—Ag2	114.1 (3)
C42—C43—H43	119.6	C35—P2—Ag2	114.0 (3)
C43—C44—C45	119.1 (9)	C23—P2—Ag2	113.3 (3)
C43—C44—H44	120.5	P1—Ag1—N3	137.1 (2)
C45—C44—H44	120.5	P1—Ag1—N2	121.37 (17)
C46—C45—C44	119.8 (9)	N3—Ag1—N2	84.7 (2)
C46—C45—H45	120.1	P1—Ag1—N1	121.91 (16)
C44—C45—H45	120.1	N3—Ag1—N1	84.0 (2)
C41—C46—C45	121.0 (9)	N2—Ag1—N1	95.5 (2)
C41—C46—H46	119.5	P1—Ag1—Ag2	128.18 (6)
C45—C46—H46	119.5	N3—Ag1—Ag2	94.7 (2)
C48—C47—C52	120.0	N2—Ag1—Ag2	49.31 (18)
C48—C47—P3	117.4 (10)	N1—Ag1—Ag2	48.87 (14)
C52—C47—P3	122.5 (10)	N4—Ag2—P2	137.2 (2)
C47—C48—C49	120.0	N4—Ag2—N1	84.6 (2)
C47—C48—H48	120.0	P2—Ag2—N1	123.30 (16)
C49—C48—H48	120.0	N4—Ag2—N2	85.1 (2)
C50—C49—C48	120.0	P2—Ag2—N2	119.82 (16)
C50—C49—H49	120.0	N1—Ag2—N2	94.5 (2)
C48—C49—H49	120.0	N4—Ag2—Ag1	95.2 (2)
C49—C50—C51	120.0	P2—Ag2—Ag1	127.53 (6)
C49—C50—H50	120.0	N1—Ag2—Ag1	48.85 (16)
C51—C50—H50	120.0	N2—Ag2—Ag1	48.24 (14)
C52—C51—C50	120.0	P3—Ag3—N4	121.2 (6)
C52—C51—H51	120.0	P3—Ag3—N1	134.0 (6)
C50—C51—H51	120.0	N4—Ag3—N1	84.2 (3)
C51—C52—C47	120.0	P3—Ag3—N3	126.6 (5)
C51—C52—H52	120.0	N4—Ag3—N3	95.5 (3)
C47—C52—H52	120.0	N1—Ag3—N3	82.2 (3)
C54—C53—C58	120.0	P3—Ag3—Ag4	132.9 (6)
C54—C53—P3	117.5 (9)	N4—Ag3—Ag4	52.4 (3)
C58—C53—P3	122.3 (9)	N1—Ag3—Ag4	93.0 (3)
C55—C54—C53	120.0	N3—Ag3—Ag4	46.0 (2)
C55—C54—H54	120.0	C41—P3—C53	101.4 (11)
C53—C54—H54	120.0	C41—P3—C47	108.6 (10)
C56—C55—C54	120.0	C53—P3—C47	104.2 (12)
C56—C55—H55	120.0	C41—P3—Ag3	113.8 (10)
C54—C55—H55	120.0	C53—P3—Ag3	112.9 (9)
C55—C56—C57	120.0	C47—P3—Ag3	114.7 (10)
C55—C56—H56	120.0	C71—P4—C65	104.4 (10)
C57—C56—H56	120.0	C71—P4—C59	101.9 (8)
C58—C57—C56	120.0	C65—P4—C59	112.4 (8)
C58—C57—H57	120.0	C71—P4—Ag4	111.0 (6)
C56—C57—H57	120.0	C65—P4—Ag4	113.3 (8)
C57—C58—C53	120.0	C59—P4—Ag4	112.9 (7)
C57—C58—H58	120.0	N2—Ag4—N3	86.8 (3)
C53—C58—H58	120.0	N2—Ag4—P4	140.2 (5)
C48'—C47'—C52'	120.0	N3—Ag4—P4	121.2 (5)
C48'—C47'—P3'	116.0 (12)	N2—Ag4—N4	82.1 (3)
C52'—C47'—P3'	124.0 (12)	N3—Ag4—N4	93.1 (3)
C47'—C48'—C49'	120.0	P4—Ag4—N4	119.9 (3)
C47'—C48'—H48'	120.0	N2—Ag4—Ag3	94.6 (3)
C49'—C48'—H48'	120.0	N3—Ag4—Ag3	49.2 (3)
C48'—C49'—C50'	120.0	P4—Ag4—Ag3	124.9 (4)
C48'—C49'—H49'	120.0	N4—Ag4—Ag3	46.6 (2)
C50'—C49'—H49'	120.0	C47'—P3'—C53'	106.4 (14)
C51'—C50'—C49'	120.0	C47'—P3'—C41	103.4 (11)
C51'—C50'—H50'	120.0	C53'—P3'—C41	106.0 (13)
C49'—C50'—H50'	120.0	C47'—P3'—Ag3'	111.5 (12)
C52'—C51'—C50'	120.0	C53'—P3'—Ag3'	116.9 (10)
C52'—C51'—H51'	120.0	C41—P3'—Ag3'	111.6 (11)
C50'—C51'—H51'	120.0	N1—Ag3'—N3	85.9 (4)
C51'—C52'—C47'	120.0	N1—Ag3'—P3'	137.2 (6)
C51'—C52'—H52'	120.0	N3—Ag3'—P3'	120.0 (6)
C47'—C52'—H52'	120.0	N1—Ag3'—N4	84.6 (3)
C54'—C53'—C58'	120.0	N3—Ag3'—N4	97.1 (3)
C54'—C53'—P3'	117.2 (11)	P3'—Ag3'—N4	120.9 (6)
C58'—C53'—P3'	122.7 (11)	N1—Ag3'—Ag4'	95.7 (3)
C55'—C54'—C53'	120.0	N3—Ag3'—Ag4'	53.5 (3)
C55'—C54'—H54'	120.0	P3'—Ag3'—Ag4'	127.0 (6)
C53'—C54'—H54'	120.0	N4—Ag3'—Ag4'	46.1 (3)
C54'—C55'—C56'	120.0	C65'—P4'—C59	102.3 (9)
C54'—C55'—H55'	120.0	C65'—P4'—C71'	104.4 (11)
C56'—C55'—H55'	120.0	C59—P4'—C71'	107.0 (9)
C55'—C56'—C57'	120.0	C65'—P4'—Ag4'	113.2 (9)
C55'—C56'—H56'	120.0	C59—P4'—Ag4'	115.3 (8)
C57'—C56'—H56'	120.0	C71'—P4'—Ag4'	113.5 (7)
C58'—C57'—C56'	120.0	N4—Ag4'—P4'	122.0 (4)
C58'—C57'—H57'	120.0	N4—Ag4'—N2	88.6 (4)
C56'—C57'—H57'	120.0	P4'—Ag4'—N2	138.5 (5)
C57'—C58'—C53'	120.0	N4—Ag4'—N3	95.8 (3)
C57'—C58'—H58'	120.0	P4'—Ag4'—N3	118.5 (5)
C53'—C58'—H58'	120.0	N2—Ag4'—N3	80.9 (3)
C64—C59—C60	119.1 (10)	N4—Ag4'—Ag3'	50.5 (3)
C64—C59—P4	122.2 (9)	P4'—Ag4'—Ag3'	126.0 (5)
C60—C59—P4	118.3 (9)	N2—Ag4'—Ag3'	94.7 (3)
C64—C59—P4'	121.5 (9)	N3—Ag4'—Ag3'	47.8 (3)
C60—C59—P4'	118.5 (9)		
			
C10—C5—C6—C7	−0.3 (14)	C74'—C75'—C76'—C71'	0.0
P1—C5—C6—C7	178.4 (8)	C72'—C71'—C76'—C75'	0.0
C5—C6—C7—C8	1.8 (16)	P4'—C71'—C76'—C75'	−177.9 (13)
C6—C7—C8—C9	−2.7 (17)	C12—C11—P1—C5	−11 (3)
C7—C8—C9—C10	2.3 (16)	C16—C11—P1—C5	174.1 (15)
C8—C9—C10—C5	−0.9 (15)	C12—C11—P1—C17	−122 (3)
C6—C5—C10—C9	−0.1 (13)	C16—C11—P1—C17	62.6 (16)
P1—C5—C10—C9	−178.7 (7)	C12—C11—P1—C11'	−163 (16)
C16—C11—C12—C13	0.0	C16—C11—P1—C11'	22 (14)
P1—C11—C12—C13	−175 (3)	C12—C11—P1—Ag1	111 (3)
C11—C12—C13—C14	0.0	C16—C11—P1—Ag1	−64.3 (17)
C12—C13—C14—C15	0.0	C6—C5—P1—C11	80.9 (13)
C13—C14—C15—C16	0.0	C10—C5—P1—C11	−100.5 (13)
C14—C15—C16—C11	0.0	C6—C5—P1—C17	−168.2 (7)
C12—C11—C16—C15	0.0	C10—C5—P1—C17	10.5 (9)
P1—C11—C16—C15	175 (2)	C6—C5—P1—C11'	83.7 (9)
C16'—C11'—C12'—C13'	0.0	C10—C5—P1—C11'	−97.6 (9)
P1—C11'—C12'—C13'	175.1 (10)	C6—C5—P1—Ag1	−41.7 (8)
C11'—C12'—C13'—C14'	0.0	C10—C5—P1—Ag1	137.0 (7)
C12'—C13'—C14'—C15'	0.0	C22—C17—P1—C11	−144.3 (14)
C13'—C14'—C15'—C16'	0.0	C18—C17—P1—C11	34.5 (14)
C14'—C15'—C16'—C11'	0.0	C22—C17—P1—C5	108.6 (8)
C12'—C11'—C16'—C15'	0.0	C18—C17—P1—C5	−72.7 (8)
P1—C11'—C16'—C15'	−175.2 (10)	C22—C17—P1—C11'	−140.4 (8)
C22—C17—C18—C19	2.6 (13)	C18—C17—P1—C11'	38.4 (9)
P1—C17—C18—C19	−176.2 (7)	C22—C17—P1—Ag1	−17.3 (9)
C17—C18—C19—C20	−1.7 (15)	C18—C17—P1—Ag1	161.5 (6)
C18—C19—C20—C21	−0.5 (19)	C12'—C11'—P1—C11	45 (14)
C19—C20—C21—C22	2 (2)	C16'—C11'—P1—C11	−140 (15)
C18—C17—C22—C21	−1.4 (16)	C12'—C11'—P1—C5	16.9 (12)
P1—C17—C22—C21	177.5 (9)	C16'—C11'—P1—C5	−167.9 (10)
C20—C21—C22—C17	−0.8 (19)	C12'—C11'—P1—C17	−94.9 (12)
C28—C23—C24—C25	−0.7 (13)	C16'—C11'—P1—C17	80.3 (10)
P2—C23—C24—C25	−179.5 (7)	C12'—C11'—P1—Ag1	141.8 (12)
C23—C24—C25—C26	−0.5 (15)	C16'—C11'—P1—Ag1	−43.0 (11)
C24—C25—C26—C27	0.5 (17)	C34—C29—P2—C35	−93.6 (10)
C25—C26—C27—C28	0.6 (18)	C30—C29—P2—C35	86.1 (8)
C26—C27—C28—C23	−1.8 (17)	C34—C29—P2—C23	17.7 (10)
C24—C23—C28—C27	1.8 (15)	C30—C29—P2—C23	−162.6 (7)
P2—C23—C28—C27	−179.3 (8)	C34—C29—P2—Ag2	141.3 (9)
C34—C29—C30—C31	−0.6 (13)	C30—C29—P2—Ag2	−39.0 (8)
P2—C29—C30—C31	179.7 (7)	C40—C35—P2—C29	41.2 (9)
C29—C30—C31—C32	0.1 (13)	C36—C35—P2—C29	−139.9 (8)
C30—C31—C32—C33	−1.5 (17)	C40—C35—P2—C23	−68.0 (9)
C31—C32—C33—C34	3 (2)	C36—C35—P2—C23	110.9 (8)
C30—C29—C34—C33	2.5 (18)	C40—C35—P2—Ag2	166.5 (7)
P2—C29—C34—C33	−177.8 (12)	C36—C35—P2—Ag2	−14.7 (9)
C32—C33—C34—C29	−4 (2)	C24—C23—P2—C29	−100.9 (8)
C40—C35—C36—C37	−1.8 (15)	C28—C23—P2—C29	80.3 (8)
P2—C35—C36—C37	179.3 (9)	C24—C23—P2—C35	9.0 (9)
C35—C36—C37—C38	−0.9 (18)	C28—C23—P2—C35	−169.8 (7)
C36—C37—C38—C39	2.3 (18)	C24—C23—P2—Ag2	134.9 (7)
C37—C38—C39—C40	−1.1 (17)	C28—C23—P2—Ag2	−43.9 (8)
C38—C39—C40—C35	−1.6 (15)	C46—C41—P3—C53	148.6 (10)
C36—C35—C40—C39	3.0 (14)	C42—C41—P3—C53	−37.2 (14)
P2—C35—C40—C39	−178.1 (7)	P3'—C41—P3—C53	39 (7)
C46—C41—C42—C43	−2.7 (15)	C46—C41—P3—C47	−102.0 (13)
P3—C41—C42—C43	−176.7 (10)	C42—C41—P3—C47	72.1 (14)
P3'—C41—C42—C43	171.9 (11)	P3'—C41—P3—C47	148 (8)
C41—C42—C43—C44	1.6 (15)	C46—C41—P3—Ag3	27.1 (13)
C42—C43—C44—C45	0.8 (15)	C42—C41—P3—Ag3	−158.8 (8)
C43—C44—C45—C46	−2.0 (14)	P3'—C41—P3—Ag3	−83 (7)
C42—C41—C46—C45	1.4 (15)	C54—C53—P3—C41	−79.2 (13)
P3—C41—C46—C45	175.9 (10)	C58—C53—P3—C41	95.2 (13)
P3'—C41—C46—C45	−173.1 (11)	C54—C53—P3—C47	168.0 (11)
C44—C45—C46—C41	0.9 (15)	C58—C53—P3—C47	−17.5 (16)
C52—C47—C48—C49	0.0	C54—C53—P3—Ag3	42.9 (15)
P3—C47—C48—C49	−177.3 (14)	C58—C53—P3—Ag3	−142.6 (11)
C47—C48—C49—C50	0.0	C48—C47—P3—C41	169.8 (11)
C48—C49—C50—C51	0.0	C52—C47—P3—C41	−7.4 (16)
C49—C50—C51—C52	0.0	C48—C47—P3—C53	−82.8 (14)
C50—C51—C52—C47	0.0	C52—C47—P3—C53	100.0 (13)
C48—C47—C52—C51	0.0	C48—C47—P3—Ag3	41.2 (15)
P3—C47—C52—C51	177.2 (15)	C52—C47—P3—Ag3	−136.1 (10)
C58—C53—C54—C55	0.0	C72—C71—P4—C65	1.4 (13)
P3—C53—C54—C55	174.6 (15)	C76—C71—P4—C65	−176.6 (10)
C53—C54—C55—C56	0.0	C72—C71—P4—C59	118.5 (11)
C54—C55—C56—C57	0.0	C76—C71—P4—C59	−59.5 (11)
C55—C56—C57—C58	0.0	C72—C71—P4—Ag4	−121.1 (10)
C56—C57—C58—C53	0.0	C76—C71—P4—Ag4	60.9 (11)
C54—C53—C58—C57	0.0	C66—C65—P4—C71	102.2 (12)
P3—C53—C58—C57	−174.3 (16)	C70—C65—P4—C71	−79.9 (12)
C52'—C47'—C48'—C49'	0.0	C66—C65—P4—C59	−7.4 (14)
P3'—C47'—C48'—C49'	−179.0 (17)	C70—C65—P4—C59	170.4 (10)
C47'—C48'—C49'—C50'	0.0	C66—C65—P4—Ag4	−136.8 (10)
C48'—C49'—C50'—C51'	0.0	C70—C65—P4—Ag4	41.0 (12)
C49'—C50'—C51'—C52'	0.0	C64—C59—P4—C71	−48.3 (12)
C50'—C51'—C52'—C47'	0.0	C60—C59—P4—C71	138.0 (10)
C48'—C47'—C52'—C51'	0.0	P4'—C59—P4—C71	43 (3)
P3'—C47'—C52'—C51'	178.9 (18)	C64—C59—P4—C65	62.9 (13)
C58'—C53'—C54'—C55'	0.0	C60—C59—P4—C65	−110.7 (12)
P3'—C53'—C54'—C55'	−178.3 (16)	P4'—C59—P4—C65	155 (4)
C53'—C54'—C55'—C56'	0.0	C64—C59—P4—Ag4	−167.4 (8)
C54'—C55'—C56'—C57'	0.0	C60—C59—P4—Ag4	18.9 (12)
C55'—C56'—C57'—C58'	0.0	P4'—C59—P4—Ag4	−76 (3)
C56'—C57'—C58'—C53'	0.0	C48'—C47'—P3'—C53'	−82.6 (16)
C54'—C53'—C58'—C57'	0.0	C52'—C47'—P3'—C53'	98.5 (16)
P3'—C53'—C58'—C57'	178.2 (17)	C48'—C47'—P3'—C41	166.0 (12)
C64—C59—C60—C61	−0.3 (18)	C52'—C47'—P3'—C41	−13.0 (17)
P4—C59—C60—C61	173.6 (10)	C48'—C47'—P3'—Ag3'	45.9 (16)
P4'—C59—C60—C61	−169.8 (10)	C52'—C47'—P3'—Ag3'	−133.0 (12)
C59—C60—C61—C62	1.2 (18)	C54'—C53'—P3'—C47'	159.2 (12)
C60—C61—C62—C63	−1.6 (17)	C58'—C53'—P3'—C47'	−19.1 (19)
C61—C62—C63—C64	1.1 (17)	C54'—C53'—P3'—C41	−91.3 (13)
C62—C63—C64—C59	−0.2 (16)	C58'—C53'—P3'—C41	90.5 (16)
C60—C59—C64—C63	−0.2 (16)	C54'—C53'—P3'—Ag3'	33.9 (17)
P4—C59—C64—C63	−173.9 (9)	C58'—C53'—P3'—Ag3'	−144.3 (12)
P4'—C59—C64—C63	169.0 (10)	C46—C41—P3'—C47'	−108.6 (13)
C70—C65—C66—C67	0.0	C42—C41—P3'—C47'	77.0 (14)
P4—C65—C66—C67	177.8 (14)	P3—C41—P3'—C47'	−33 (7)
C65—C66—C67—C68	0.0	C46—C41—P3'—C53'	139.7 (12)
C66—C67—C68—C69	0.0	C42—C41—P3'—C53'	−34.7 (15)
C67—C68—C69—C70	0.0	P3—C41—P3'—C53'	−145 (8)
C68—C69—C70—C65	0.0	C46—C41—P3'—Ag3'	11.4 (15)
C66—C65—C70—C69	0.0	C42—C41—P3'—Ag3'	−163.1 (8)
P4—C65—C70—C69	−177.9 (13)	P3—C41—P3'—Ag3'	87 (7)
C76—C71—C72—C73	0.0	C66'—C65'—P4'—C59	−9.1 (15)
P4—C71—C72—C73	−178.0 (12)	C70'—C65'—P4'—C59	172.0 (10)
C71—C72—C73—C74	0.0	C66'—C65'—P4'—C71'	102.3 (14)
C72—C73—C74—C75	0.0	C70'—C65'—P4'—C71'	−76.6 (12)
C73—C74—C75—C76	0.0	C66'—C65'—P4'—Ag4'	−133.8 (11)
C74—C75—C76—C71	0.0	C70'—C65'—P4'—Ag4'	47.3 (13)
C72—C71—C76—C75	0.0	C64—C59—P4'—C65'	70.0 (13)
P4—C71—C76—C75	178.0 (12)	C60—C59—P4'—C65'	−120.7 (12)
C70'—C65'—C66'—C67'	0.0	P4—C59—P4'—C65'	−27 (3)
P4'—C65'—C66'—C67'	−178.9 (16)	C64—C59—P4'—C71'	−39.4 (13)
C65'—C66'—C67'—C68'	0.0	C60—C59—P4'—C71'	129.8 (11)
C66'—C67'—C68'—C69'	0.0	P4—C59—P4'—C71'	−137 (4)
C67'—C68'—C69'—C70'	0.0	C64—C59—P4'—Ag4'	−166.7 (8)
C68'—C69'—C70'—C65'	0.0	C60—C59—P4'—Ag4'	2.6 (13)
C66'—C65'—C70'—C69'	0.0	P4—C59—P4'—Ag4'	96 (3)
P4'—C65'—C70'—C69'	179.0 (15)	C72'—C71'—P4'—C65'	−14.6 (14)
C76'—C71'—C72'—C73'	0.0	C76'—C71'—P4'—C65'	163.2 (11)
P4'—C71'—C72'—C73'	177.7 (14)	C72'—C71'—P4'—C59	93.3 (13)
C71'—C72'—C73'—C74'	0.0	C76'—C71'—P4'—C59	−88.8 (13)
C72'—C73'—C74'—C75'	0.0	C72'—C71'—P4'—Ag4'	−138.3 (11)
C73'—C74'—C75'—C76'	0.0	C76'—C71'—P4'—Ag4'	39.5 (15)

### Hydrogen-bond geometry (Å, º)

**Table:**

*D*—H···*A*	*D*—H	H···*A*	*D*···*A*	*D*—H···*A*
C9—H9···O2^i^	0.93	2.37	3.177 (12)	145
C16′—H16′···O2	0.93	2.59	3.324 (17)	136
C25—H25···O1^ii^	0.93	2.48	3.358 (12)	157
C51—H51···O4^iii^	0.93	2.22	3.07 (2)	151
C67—H67···O3^iv^	0.93	2.19	3.01 (2)	147

Symmetry codes: (i) −*y*+1, *x*, −*z*+2; (ii) −*y*+1, *x*−1, −*z*+2; (iii) *y*+1, −*x*+1, −*z*+1; (iv) *y*, −*x*+1, −*z*+1.
